# What does our region need in order to strengthen public policies on sugar-sweetened beverages? decision-makers’ dialogue

**DOI:** 10.17843/rpmesp.2023.401.12394

**Published:** 2023-03-31

**Authors:** Andrea Alcaraz, Lucas Perelli, María Belén Rodriguez, Alfredo Palacios, Ariel Bardach, Kimberly-Ann Gittens-Baynes, Cid Vianna, Giovanni Guevara, Sebastián García-Martí, Agustín Ciapponi, Federico Augustovski, María Belizán, Andrés Pichon-Riviere

**Affiliations:** 1 Instituto de Efectividad Clínica y Sanitaria (IECS), Argentina. Instituto de Efectividad Clínica y Sanitaria (IECS) Argentina; 2 Consejo Nacional de Investigaciones Científicas y Técnicas (CONICET), Argentina. Consejo Nacional de Investigaciones Científicas y Técnicas Consejo Nacional de Investigaciones Científicas y Técnicas (CONICET) Argentina; 3 The University of the West Indies, HEU, Centre for Health Economics,(UWI-HEU), St. Augustine Campus, Trinidad and Tobago. The University of the West Indies, HEU Centre for Health Economics, (UWI-HEU) St. Augustine Campus Trinidad and Tobago; 4 Universidade Federal do Rio de Janeiro, Rio de Janeiro, Brasil. Universidade Federal do Rio de Janeiro Universidade Federal do Rio de Janeiro Rio de Janeiro Brazil; 5 Universidad Católica de El Salvador, El Salvador, El Salvador. Universidad Católica de El Salvador El Salvador El Salvador; 6 School of Public Health, Faculty of Medicine, Universidad de Buenos Aires (UBA), Buenos Aires, Argentina Universidad de Buenos Aires School of Public Health Faculty of Medicine Universidad de Buenos Aires (UBA) Buenos Aires Argentina

**Keywords:** Sugar-Sweetened Beverages, Noncommunicable Diseases, Public Policy, Decision Making Qualitative Research

## Abstract

In order to prioritize public policies to reduce the consumption of sugar-sweetened beverages in Argentina, Brazil, El Salvador and Trinidad and Tobago and to identify information gaps related to the burden of disease attributable to their consumption, a policy dialogue was held with government members, civil society organizations, researchers and communicators from Latin American and Caribbean countries. Presentations and deliberative workshops were conducted using semi-structured data collection tools and group discussions. The prioritized interventions were tax increases, front labeling, restriction of advertising, promotion and sponsorship, and modifications regarding the school environment. The main perceived barrier was the interference from the food industry. This dialogue among decision-makers led to the identification of priority public policies to reduce the consumption of sugar-sweetened beverages in the region.

## INTRODUCTION

Globally, noncommunicable diseases (NCDs) are the leading cause of mortality and most deaths occur in low- and middle-income countries ^1^. In addition to the significant burden of disease, NCDs also place a significant economic burden on health systems and society, which can be a barrier to global development ^2^.

The burden of disease and mortality due to noncommunicable diseases is closely linked to overweight and obesity, with poor diet and sedentary lifestyle being the main risk factors. Sugar-sweetened beverages (SSBs) are a major source of added sugars in the diet, with high caloric content and low nutritional value; their consumption is associated with weight gain and the risk of developing type 2 diabetes and other related conditions ^3^^-^^6^. Its consumption in Latin America is high when compared to other regions, with an average consumption of 370 ml per day compared to the world average of 140 ml ^7^. For this reason, in recent years, different public policies have been developed aiming to reduce SSB consumption, such as fiscal policies, labeling, restrictions on advertising, promotion and sponsorship ^8^^-^^11^.

Identifying the best available evidence to support the creation of public health policies is becoming increasingly relevant in the international agenda with the aim of strengthening healthcare systems worldwide. However, there are significant barriers to address the gap between knowledge and its appropriate use for the benefit of society, which can lead to ineffective or even harmful public policies ^12^. Policy dialogues are useful tools to guide the creation of public policies, allowing interaction between the most relevant actors (researchers, decision-makers, communicators and civil society), as well as the integration of explicit and tacit knowledge in an inclusive and transparent environment, providing the opportunity to reflect on the application of evidence in different contexts with implications for the final decision making ^13^. 

This article presents the results of a policy dialogue involving government members, civil society organizations, researchers and communicators from four countries in Latin America and the Caribbean (Argentina, Brazil, El Salvador and Trinidad and Tobago) on policies for the control of alcohol consumption. It was carried out within the framework of a project funded by Canada’s International Development Research Centre (IDRC) entitled “Empowering health decision-makers to achieve regional objectives in sugar-sweetened beverage control policies in Latin America and the Caribbean: building a framework for assessing the burden of disease, the cost-effectiveness of available interventions and estimating the burden of disease in four countries”. The aim of the project was to develop a simulation model to estimate the burden of disease attributable to SSB consumption in the four countries and to evaluate the cost-effectiveness of interventions for its control ^14^. The policy dialogue described here was carried out to inform the development of key aspects of the model (such as the target population and the outcomes) and to gain a better understanding of the problem at the regional level. In turn, the decision-makers’ dialogue aimed to identify the public policy interventions prioritized worldwide for the control of SSB consumption and the real or perceived barriers as well as the facilitators in order to implement new interventions or reinforce those already implemented; and to understand the information needs regarding the promotion and simplification of decision making.

## METHODS

Quali-quantitative study describing the main findings of the policy dialogue conducted through two face-to-face workshops in Buenos Aires, Argentina on May 14 and 15, 2018.

### Participants

Participant selection was carried out with the intention of including government members of the Ministries of Health and Finance/Economy, members of civil organizations dedicated to public health with a background in actions aimed at the control of SSB consumption, researchers or academics who had participated or were participating in or had published studies related to the control of SSB consumption in the four countries. We decided to include members of the Pan American Health Organization (PAHO) and the United Nations Children’s Emergency Fund (UNICEF) as regional organizations. The coordinating team based in Argentina included 11 researchers from the partner countries, with a project leader for Brazil, El Salvador and Trinidad and Tobago.

Researchers had experience in research on burden of disease, economic evaluations and/or public policy. The team consisted of physicians (n=6), social scientists (n=3), health economists (n=2) and research fellows (n=2). The project was designed with an active gender perspective to achieve representativeness of both sexes, all four countries and all parties involved.

### Development, conduct and program of the event

In order to conduct the policy dialogue, the working team developed: (a) a detailed program, (b) a comprehensive list of public policy interventions for the control of sugar-sweetened beverages, (c) a list of relevant outcomes to be considered when assessing the burden of disease due to sugar-sweetened beverages or the impact of interventions, and (d) four semi-structured data collection instruments that inquired about interventions for the reduction of SSB consumption, their prioritization for the Latin American and Caribbean (LAC) region, population subgroups particularly those vulnerable or impacted by the consumption of SSB, barriers and facilitators, and the need and availability of information for the development of healthy eating policies in Latin America and the Caribbean.

Two deliberative workshops were held as part of the policy dialogue. The participants were initially divided into three groups (A, B and C), ensuring that in each group all the sectors and at least three of the four countries were represented. Afterwards, the work of each group was presented to all participants.

The first day began with a presentation of the objectives of the deliberative dialogue, then a regional expert gave a presentation on the status of SSB consumption in LAC, which was followed by presentations given by four experts on the respective statuses of Argentina, Brazil, El Salvador and Trinidad and Tobago. The three groups were then provided with a list of possible interventions to reduce SSB consumption, which they were asked to prioritize. Subsequently, the barriers and facilitators for the implementation and success of these interventions in each of the countries were assessed.

On the second day, the second deliberative workshop was held to inquire about the needs and availability of information regarding the promotion of policies to reduce SSB consumption. Subsequently, a country-specific activity was held to discuss the availability of epidemiological and economic information at the local level. All findings from the deliberative workshops and the country-specific activity were shared with the rest of the participants at the end of the day.

### Data analysis

We developed an analysis matrix that included the dimensions outlined in the objectives of the policy dialogue and the data collection instruments. Two recorders were used to register the group sessions, and we used the transcripts for the analysis. These data were analyzed by two researchers and then organized in tables to facilitate their reading.

### Ethics

Oral informed consent was obtained from all participants. Participants were informed that the audio recordings of the group sessions would be transcribed to complement the data collection.

## RESULTS

The main characteristics of the participants are presented in Table 1.

Our findings are described below for each dimension of the thematic analysis.

**Table 1:** Characteristics of the participants.

Characteristic	n (%)
Number of participants	
Focal group A	7/25 (28.0)
Focal group B	8/25 (32.0)
Focal group C	10/25 (40.0)
Ratio of women ^a^	
Focal group A	3/7 (43.0)
Focal group B	4/8 (50.0)
Focal group C	6/10 (60.0)
Role of participants	
Academic	6/25 (24.0)
Decision-maker	9/25 (36.0)
Civil society	7/25 (28.0)
Another	3/25 (12.0)
Country of origin	
Argentina	11/25 (44.0)
Brazil	4/25 (16.0)
El Salvador	4/25 (16.0)
Trinidad and Tobago	3/25(12.0)
Another	3/25 (12.0)

a The denominator for all percentages is 25, except in the case of the ratio of women, which varies in each focus group, which is why the proportion of women is greater than 100%.

### Consumption situations in each country

Participants associated the use of SSB with festive events, noting that these events are very frequent, therefore, there are many moments to consume SSBs over the course of a year. Other participants identified frequent SSB consumption in adults and children as part of daily life, and highlighted the great accessibility that exists in environments such as school, clubs or home.

### Prioritized interventions

Through an exhaustive review of the literature and the contribution made by the experts who participated in the deliberative workshops, 89 potentially implementable interventions were identified in Latin American and Caribbean countries. These interventions were classified into four groups: a) policies to modify the price of sugar-sweetened beverages (taxes) or consumption alternatives (subsidies for sugar-free beverages or water); b) graphic labeling in the front face of food packaging; c) regulation and/or prohibition of direct and indirect advertising, promotion and sponsorship of sugar-sweetened beverages; d) educational actions and actions to modify the school environment (public and private).

The implementation of policies to modify the price of SSB via taxes was prioritized in all three groups, with special emphasis on taxing the amount of sugar and the volume of SSB. Some of the important reasons for prioritizing these measures were the amount of evidence about their benefits and the potential use of these funds to address health problems derived from SSB consumption in the population.

Likewise, graphic front-of-package food labeling was also prioritized by the three groups, explicitly mentioning the need to have large, graphic warnings with direct and clear interpretation on the front of food packages. The participants highlighted the favorable local and regional socio-political context that encourages collaboration between countries for the implementation of this measure, the existence of ongoing regional experiences, and stressed the importance of this measure as the one that “will trigger the implementation of the rest of the interventions” by identifying which products should be included in the other interventions.

Restrictions on advertising and marketing of SSBs were also prioritized by the three groups, despite recognizing that the evidence is scarce regarding the effect of prohibition policies. The scope of potential regulatory and/or prohibition measures were an important concern for the three groups, suggesting that it should reach not only mass media, but also the Internet, mobile applications and social networks.

Finally, measures to modify the school environment were prioritized by only two of the groups, and educational measures by only one. Some of the reasons for prioritizing measures aimed at modifying the school environment were: short-term execution, the importance of the measure in responding to the Declaration of the Rights of the Child, and the fact that it had already been implemented in some countries in the region, such as Brazil, where only water and natural juices were allowed to be sold.

### Barriers and facilitators for the implementation of public policies

The three groups considered that the barriers presented in Table 2 threatened the implementation of interventions to control SSB consumption. Four types of barriers were identified for general measures: political advocacy or “lobbying” by the industries producing SSB, potential social rejection of the measures, difficulty in political articulation, and difficulties in implementation and monitoring. The barriers related specific measures are also described.

**Table 2:** Participants’ perceptions of the barriers that threaten the implementation of interventions to control sugar-sweetened beverage consumption in Argentina, Brazil, El Salvador and Trinidad and Tobago.

Advocacy or “lobbying” by the sugar beverage industries
Lobbying activities focused on congressmen that hinder and/or delay the enactment of regulatory standards.
Private interests of political decision makers not to implement measures that affect the funding of their political campaigns.
Sponsorship activities for schools or sporting events in which children and adolescents participate.
Social responsibility programs by the manufacturing companies that negatively influence public opinion.
Promotion of self-regulation programs of the producing industries despite the fact that it is known that they have little or no impact.
Opposition of “interested parties” / social rejection
Coffee shop/kiosk owners; school administration and “cooperatives”, who benefit from school sales.
Families (feeling of “losing control”). Lack of health risk perception.
Consumer perception that certain measures are implemented “to lower costs”.
Popular resistance to the taxation of certain mass consumption products due to distrust about the destination of the proceeds.
Perception that regional economies will be threatened / loss of jobs.
Potential pressure from industries that provide inputs for the commercialization of sugar-sweetened beverages.
Difficult political articulation
Relevant for federal countries, where it is necessary for jurisdictions to ratify national legislation.
Perception of poor interministerial coordination, mainly between the Ministries of Health, Finance/Economy, Social Development and Education.
Lack of transparency in political decision-making processes.
Lack of knowledge from the Ministries of Health about the influences acting on the Ministries of Finance/Economy.
Regulations on the regional marketing of sugar-sweetened beverages, such as the Common Market of the South, which are used by the industry as arguments against modifying labels to include graphic warnings.
Lack of support from scientific societies as actors within the health system.
Difficult implementation, monitoring and evaluation
Public communication is challenging given the negative popular perception of regulatory measures by states.
Lack of economic resources to guarantee public access to drinking water to replace sugar-sweetened beverages.
Lack of economic and/or human resources to monitor and evaluate these measures.
Lack of scientific evidence to support the clinical and economic benefit of implementing these measures.
Disagreement among interested parties on the products to which these measures would apply.
Potential threats to specific measures
Strategies of the manufacturing companies to avoid transferring taxes to the final price.
Potential reduction in tax benefit if long-term affordability is not considered in inflationary contexts.
Cross-product demand if the scope of the measures is partial (replacement of consumption by another unhealthy product).
Multiplicity and ultra-fast modernization of communication platforms and Internet access make it difficult to regulate and/or prohibit advertising.

Regarding facilitators, the successful implementation of all the prioritized interventions in countries of the region such as Chile (frontal labeling) and Mexico (taxes) was highlighted, as well as the fact that legislative projects in some countries are already being discussed aiming to work towards these interventions, the political declaration stating the fact that obesity is a global and local epidemiological emergency, and the importance that this topic has had in the global public policy agenda, the positive experience with some of these measures when they were implemented to reduce tobacco consumption, allowing the reuse of the circuits and processes that allowed the implementation of these measures, facilitating multisectoral articulation. In all cases, the role of civil organizations as social engines to promote these interventions was highlighted. The following were considered to be important actors: scientific societies, organizations that promote political advocacy on issues related to public health, consumer law organizations, journalists and social communicators.

### Information needs of decision-makers to promote public policies

The three groups agreed on the need for evidence on the impact of these interventions in terms of: a) prevalence of obesity and overweight, diabetes, mortality and number of years of life lost prematurely; b) health impact of cancers associated with increased risk due to the consumption of AD and associated cardiovascular diseases; and c) cost to the health system. Regarding the population subgroups of interest, all groups agreed with the disaggregation of these outcomes by sex, age groups, and socioeconomic level. In the case of economic outcomes, participants mentioned that they would like to have information regarding the attributable costs by health subsystem and by health event. Some groups reported that the following outcomes were relevant: prevalence of caries, bullying, quality of life issues, health and gender inequities associated with obesity and overweight, unreimbursed health care expenditures, macroeconomic externalities, and use of health resources (hospitalizations, outpatient appointments, etc.).

### Availability and quality of the information required for the economic models and the definition of policies

The availability of information for the design of the model of burden of disease due to consumption of SSBs and of the evaluation of interventions to reduce its consumption was identified by a group discussion of representatives from each country (Table 3). Overall, participants agreed on the availability of information on basic demographic parameters and consumption data, although they noted lower availability of information about the effectiveness of interventions to reduce consumption of SSBs.

**Table 3:** Availability of information sources by country.

Type of data	Argentina	Brazil	El Salvador	Trinidad and Tobago
Cardiovascular risk factors	Yes	Yes	Yes	Yes
Consumption of sugar-sweetened beverages	Yes	Yes	To be confirmed	Yes
Demography	Yes	Yes	Probably	Yes
Epidemiology of related diseases	Partial	Yes	Yes	No
Loss of productivity	Partial	Yes	To be confirmed	Unknown
Caries	Yes	Unknown	Unknown	Unknown
Bullying	Yes	Unknown	Unknown	Unknown
Effectiveness of potential interventions	No	Partial	Partial	No
Price elasticity of demand	Yes	Yes	No	No
Tax information	Partial	Yes	To be confirmed	No
Event costs	Partial	Yes	Yes	Yes
Intervention costs	Partial	Yes	No	No

### Population groups vulnerable to the consumption of sugar-sweetened beverages

Throughout both days, participants highlighted the importance of considering children and adolescents as a particularly vulnerable group. One of the main reasons was that eating habits are created at those ages, which is why companies producing sugar-sweetened beverages target their marketing campaigns at them, violating their rights with repercussions for the rest of their lives. It was also noted that it is important to obtain data by socioeconomic level in order to make equity estimates.

It should be noted that the gender perspective was not spontaneously introduced by the participants as a dimension to be considered in the group dynamics or in the plenary sessions. In order not to induce socially expected and/or politically correct answers, we avoided asking explicitly by using this expression (“gender perspective” or “gender approach”), opting instead to inquire whether the interventions or the information should consider different population groups and, if so, which ones. In response to this inquiry, priority was mostly given to classification by age (particularly children) and by socioeconomic stratum, but not by gender. One of the groups mentioned that there are not too many differences in the consumption of SSBs between men and women, nor in cases of obesity, although perhaps in the latter, women show higher indicators because they are the ones who mostly seek medical attention. In response to other more direct questions regarding women (in view of the spontaneous non-response), the participants mentioned that they were not perceived as a differentially affected population group.

## DISCUSSION

An important group of decision-makers, academics and members of civil society organizations, were able to identify and prioritize fiscal policies, graphic front-of-pack food labeling and the regulation and/or prohibition of advertising, promotion and sponsorship of SSBs as the main interventions to reduce its consumption. Educational actions and the modification of school environment were considered relevant interventions, but not prioritized due to the difficulty in their implementation. The main barrier for the implementation of measures aimed at reducing consumption of SSBs was the interference by the manufacturing companies through different strategies such as political lobbying, delaying or hindering the enactment of regulatory standards and the promotion of corporate social responsibility programs and self-regulation initiatives. This perception is aligned with multiple examples in the literature regarding food industry interference in the implementation of policies aimed at the control of noncommunicable diseases in Latin America ^15^^-^^17^. Other barriers mentioned by the participants were the difficulty in the implementation, monitoring, evaluation and articulation of policies aimed at reducing the consumption of SSBs and the need for resources to carry out these activities.

The participants agreed on the relevance of having clear information on the burden of disease in both the health and economic aspects and on the impact of interventions aimed at reducing the consumption of SSBs. We found considerable heterogeneity regarding the availability of information in Brazil, Argentina, El Salvador and Trinidad and Tobago for modeling estimates of the burden of disease associated with the consumption of SSB and interventions to reduce its consumption, except for demographic information, which further increases the relevance of having reliable estimates.

One of the most important benefits of policy dialogue events is the sense of ownership of the issue by decision-makers. Moreover, dialogue, whether formal or informal, can increase trust between decision-makers, researchers, and civil society, enabling the development of constructive engagements for society as a whole, anticipating barriers and facilitators to implementation ^18^.

These types of events are relevant for the formulation of policies aimed at combating noncommunicable diseases ^19^^,^^20^. It is important to mention the experience regarding tobacco consumption in Latin America, where the existence of multiple policy dialogues over time allowed the identification and promotion of successful policies to reduce the prevalence of tobacco consumption in recent years ^21^.

As this was a face-to-face event, the number of participants per country was low and may not represent all the interests about SSBs consumption within each country, although a special effort was made to identify country leaders; particularly in large countries such as Brazil or Argentina; different subnational actors were not represented.

This event allowed for a structured discussion favoring the exchange between actors, not only from one country, but from four countries, that will generate information on the burden of disease and the economic impact of SSBs consumption in the following stages of this project. The regional contribution provided by representatives of international organizations contributed with experiences from other countries that promoted this type of policies, also creating a sense of regional need to move forward on this issue.

In conclusion, this policy dialogue allowed the identification of the main public policy interventions that should be promoted as a priority in Argentina, Brazil, El Salvador and Trinidad and Tobago to reduce the consumption of sugar-sweetened beverages, identifying barriers and facilitators, expected results to promote their implementation, as well as available and missing information on the subject.
