# Supplementary material for: What does our region need in order to strengthen public policies on sugar-sweetened beverages? decision-makers’ dialogue
Source: Rev Peru Med Exp Salud Publica. 2023 Mar 31;40(1):86–93. doi: 10.17843/rpmesp.2023.401.12394 (PMC10953643; doi:10.17843/rpmesp.2023.401.12394)
Supplement: Translation

# Translation: ¿Qué necesita nuestra región para fortalecer políticas públicas sobre bebidas azucaradas? diálogo de decisores

**Authors:** Andrea Alcaraz, Lucas Perelli, María Belén Rodriguez, Alfredo Palacios, Ariel Bardach, Kimberly-Ann Gittens-Baynes, Cid Vianna, Giovanni Guevara, Sebastián García-Martí, Agustín Ciapponi, Federico Augustovski, María Belizán, Andrés Pichon-Riviere

**Affiliations:** 1 Instituto de Efectividad Clínica y Sanitaria (IECS), Argentina.; 2 Consejo Nacional de Investigaciones Científicas y Técnicas (CONICET), Argentina.; 3 The University of the West Indies, HEU, Centre for Health Economics,(UWI-HEU), St. Augustine Campus, Trinidad and Tobago.; 4 Universidade Federal do Rio de Janeiro, Rio de Janeiro, Brazil.; 5 Universidad Católica de El Salvador, El Salvador, El Salvador.; 6 Escuela de Salud Pública, Facultad de Medicina, Universidad de Buenos Aires (UBA), Buenos Aires, Argentina

**Keywords:** Bebidas Azucaradas, Enfermedades No transmisibles, Políticas Públicas, Toma de Decisiones, Investigación Cualitativa

## Abstract

Con el objetivo de priorizar políticas públicas para disminuir el consumo de bebidas azucaradas en Argentina, Brasil, El Salvador y Trinidad y Tobago e identificar las necesidades de información relacionadas con la carga de enfermedad atribuible a su consumo se realizó un diálogo de políticas en el que participaron miembros de gobierno, organizaciones de la sociedad civil, investigadores y comunicadores de países de Latinoamérica y el Caribe. Se llevaron a cabo exposiciones y talleres deliberativos utilizándose herramientas de recolección de datos semiestructuradas y discusiones grupales facilitadas. Las intervenciones priorizadas fueron el incremento de impuestos, el etiquetado frontal, la restricción de la publicidad, promoción y patrocinio y las modificaciones del entorno escolar. La principal barrera percibida fue la interferencia de la industria alimentaria. La realización de este diálogo de decisores permitió la identificación de las políticas públicas prioritarias para disminuir el consumo de bebidas azucaradas en la región.

## INTRODUCCIÓN

A nivel mundial, las enfermedades no transmisibles (ENT) son la principal causa de mortalidad y la mayoría de éstas se producen en países de ingresos bajos y medios ^1^. Además de la importante carga de enfermedad, las ENT también suponen una importante carga económica para los sistemas sanitarios y las sociedades en general, lo que puede suponer un obstáculo para el desarrollo mundial ^2^.

La carga de enfermedad y mortalidad atribuible a las enfermedades no transmisibles está estrechamente vinculada al sobrepeso y la obesidad, siendo la mala alimentación y el sedentarismo los principales factores de riesgo. Las bebidas azucaradas (BA) son una fuente importante de azúcares añadidos en la dieta, con alto contenido calórico y bajo valor nutricional, y su consumo está asociado al aumento de peso y al riesgo de desarrollar diabetes tipo 2 y otros trastornos relacionados ^3^^-^^6^. Su consumo en Latinoamérica es elevado con relación a otras regiones, con un promedio de consumo de 370 ml al día comparado con el promedio mundial de 140 ml ^7^. Es por esto que en los últimos años se han desarrollado diferentes políticas públicas con el fin de disminuir el consumo de BA como las políticas fiscales, el etiquetado frontal, las restricciones a la publicidad, promoción y patrocinio ^8^^-^^11^. 

La identificación de la mejor evidencia disponible para respaldar la formulación de políticas en salud pública está cobrando cada vez más relevancia en la agenda internacional con el objetivo de fortalecer los sistemas de salud a nivel global. Sin embargo, pueden existir barreras importantes para abordar la brecha entre el conocimiento y el uso apropiado del mismo en beneficio de la sociedad lo que puede dar lugar a políticas públicas ineficaces o incluso perjudiciales ^12^. Los diálogos de políticas (del inglés Policy Dialogues) son herramientas útiles para guiar la formulación de políticas públicas, permitiendo la interacción entre los actores más relevantes (investigadores, tomadores de decisiones, comunicadores y la sociedad civil), así como la integración del conocimiento explícito y tácito en un ambiente inclusivo y transparente, brindando la oportunidad de reflexionar acerca de la aplicación de la evidencia en diferentes contextos con implicancias en la toma de decisión final ^13^.

Este artículo presenta los resultados de un diálogo de políticas en el que participaron miembros de gobierno, organizaciones de la sociedad civil, investigadores y comunicadores de cuatro países de Latinoamérica y el Caribe (Argentina, Brasil, El Salvador y Trinidad y Tobago) sobre políticas para el control del consumo de BA. El mismo fue realizado en el marco de un proyecto financiado por Centro Internacional de Investigaciones para el Desarrollo de Canadá (IDRC, del inglés International Development Research Centre) denominado «Empoderando a los tomadores de decisiones de salud para lograr los objetivos regionales en políticas de control de consumo de bebidas azucaradas en América Latina y el Caribe: construyendo un marco para evaluar la carga de la enfermedad, la costo-efectividad de las intervenciones disponibles y estimar la carga de la enfermedad en cuatro países». El objetivo principal del proyecto fue desarrollar un modelo de simulación para estimar la carga de enfermedad atribuible al consumo de BA en los cuatro países y evaluar la costo-efectividad de intervenciones para su control ^14^. El diálogo de políticas aquí descrito se llevó adelante para informar el desarrollo de aspectos clave del modelo (como la población objetivo, los resultados a ser generados) y tener un mayor entendimiento de la problemática a nivel de la región. A su vez, el diálogo de decisores tuvo por propósito identificar las intervenciones de políticas públicas priorizadas a nivel mundial para el control del consumo de BA; las barreras y facilitadores reales o percibidos para implementar nuevas intervenciones o reforzar las ya implementadas; y conocer las necesidades de información para promover y facilitar la toma de decisiones. 

## MÉTODOS

Estudio cuali-cuantitativo donde se describen los principales hallazgos del diálogo de políticas llevado a cabo mediante dos jornadas presenciales en Buenos Aires, Argentina los días 14 y 15 de mayo del 2018.

### Participantes

La selección de los participantes se realizó deliberadamente con la intención de incluir formalmente miembros de gobierno de Ministerios de Salud y Hacienda/Economía, miembros de organizaciones civiles dedicadas a salud pública con antecedentes en acciones orientadas al control del consumo de BA, investigadores o académicos que hayan participado o estén participando o hayan publicado estudios relacionados al control del consumo de BA de los cuatro países del estudio. Se decidió incluir en la lista de participantes a miembros de la Organización Panamericana de la Salud (OPS) y del Fondo de las Naciones Unidas para la Infancia (UNICEF, su sigla del inglés United Nations International Children Emergency Fund) como organismos regionales. Dentro del equipo coordinador con base en Argentina participaron 11 investigadores de los países asociados, con un líder de proyecto para Brasil, El Salvador y Trinidad & Tobago. 

Los investigadores tenían experiencia en estudios de carga de enfermedad, evaluaciones económicas y/o políticas públicas quedando el equipo constituido por médicos (n=6), científicos sociales (n=3), economistas de la salud (n=2) y becarios de investigación (n=2). El proyecto fue diseñado con una mirada de género activa para lograr representatividad de ambos sexos, los cuatro países y de todas las partes interesadas. 

### Desarrollo, conducción y agenda del evento

Para la conducción del diálogo de políticas, el equipo de trabajo desarrolló: a) una agenda detallada, b) un listado exhaustivo de intervenciones políticas públicas para el control de bebidas azucaradas c) un listado de desenlaces relevantes que debían ser considerados al momento de evaluar la carga de enfermedad atribuible a las bebidas azucaradas o el impacto de las intervenciones y d) cuatro instrumentos de recolección de información semiestructurados que indagaban sobre intervenciones para la reducción del consumo de BA, su priorización para la región de Latinoamérica y el Caribe (LAC), subgrupos de población especialmente vulnerables o impactados por el consumo de BA, barreras y facilitadores y finalmente necesidad y disponibilidad de información para el desarrollo de políticas de alimentación saludable en Latinoamérica y el Caribe. 

Como parte del diálogo de políticas, se realizaron dos talleres deliberativos, para los cuales los participantes fueron distribuidos inicialmente en tres grupos (A, B y C) garantizando que en cada grupo estén representados todos los sectores convocados y al menos tres de los cuatro países del estudio y luego se expuso lo trabajado en cada uno de los grupos para todos los participantes. 

La primera jornada comenzó con la presentación de los objetivos del diálogo deliberativo, la presentación de un experto regional sobre el estado de consumo de BA en LAC, y las exposiciones de cuatro expertos sobre los respectivos estados de situación de Argentina, Brasil, El Salvador y Trinidad y Tobago. A continuación, se les facilitó a los tres grupos un listado con posibles intervenciones para reducir el consumo de BA, y que las priorizaran. Posteriormente se indagó sobre las barreras y facilitadores para la implementación y éxito de estas intervenciones en forma general para los países representados.

La segunda jornada se realizó el segundo taller deliberativo para indagar sobre las necesidades y disponibilidad de información de los participantes para promover políticas para reducir el consumo de BA. Posteriormente se realizó una actividad específica por país para dialogar sobre la disponibilidad de información epidemiológica y económica a nivel local. En todos los casos, todos los hallazgos de los talleres deliberativos y la actividad por país se pusieron en común con el resto de los participantes al finalizar la jornada respectiva.

### Análisis de los datos

Se desarrolló una matriz de análisis que incluía las dimensiones planteadas en los objetivos del diálogo de políticas y los instrumentos de recolección de información. Para el análisis se tomaron como insumo las notas tomadas por dos registradores durante las sesiones grupales y las desgrabaciones de las mismas. Estos datos fueron analizados por dos participantes del grupo de investigación y organizados en tablas para facilitar su lectura.

### Ética

Todos los participantes aceptaron participar mediante un consentimiento informado administrado en forma verbal. Se informó a los participantes que los audios de las sesiones grupales serían transcritos para complementar la recolección de información. 

## RESULTADOS

Las principales características de los participantes se resumen en la Tabla 1.

A continuación, se describen los hallazgos bajo las dimensiones consideradas para el análisis temático:

**Tabla 1:** Características de los participantes.

Característica	n (%)
Cantidad de informantes clave	
Grupo focal A	7/25 (28,0)
Grupo focal B	8/25 (32,0)
Grupo focal C	10/25 (40,0)
Proporción de mujeres ^a^	
Grupo focal A	3/7 (43,0)
Grupo focal B	4/8 (50,0)
Grupo focal C	6/10 (60,0)
Rol de los informantes clave	
Académico	6/25 (24,0)
Decisor	9/25 (36,0)
Sociedad civil	7/25 (28,0)
Otro	3/25 (12,0)
País de origen	
Argentina	11/25 (44,0)
Brasil	4/25 (16,0)
El Salvador	4/25 (16,0)
Trinidad y Tobago	3/25(12,0)
Otro	3/25 (12,0)

a El denominador para todos los porcentajes es 25, salvo en el caso de proporción de mujeres que varia en cada grupo focal, por esta razón la proporción de mujeres es mayor a 100%.

### Situaciones de consumo en cada país

Los participantes destacaron el consumo de BA asociado a eventos festivos, remarcando que estos eventos tienen una elevada frecuencia, por lo que a lo largo de un año son múltiples las situaciones de consumo. Otros participantes identificaron una elevada frecuencia de situaciones de consumo de BA en adultos y niños como parte de la vida diaria, y destacaron la gran accesibilidad que existe en entornos como la escuela, el club o el hogar.

### Intervenciones priorizadas

Mediante una revisión exhaustiva de la literatura y del aporte realizado por los expertos que participaron de los talleres deliberativos se identificaron 89 intervenciones potencialmente implementables en países de Latinoamérica y el Caribe. Dichas intervenciones fueron clasificadas en cuatro grupos: a) políticas de modificación del precio de las bebidas azucaradas (impuestos) o de alternativas de consumo (subsidios a bebidas sin azúcar o aguas); b) etiquetado gráfico frontal de alimentos; c) regulación y/o prohibición de la publicidad directa e indirecta, la promoción y el patrocinio de bebidas azucaradas; d) acciones educativas propiamente dichas y acciones de modificación del entorno escolar (ámbitos públicos y privados).

La implementación de políticas de modificación del precio de las BA vía impuestos fue priorizada en los tres grupos, con especial énfasis en el gravamen a la cantidad de azúcar y al volumen de las BA. Entre las razones para priorizar estas medidas, se destacó la cantidad de evidencia acerca de sus beneficios, el potencial uso de estos fondos para abordar los problemas de salud derivados del consumo de BA en la población.

Así mismo, el etiquetado gráfico frontal de alimentos también fue priorizado por los tres grupos indagados, mencionándose explícitamente la necesidad de contar con advertencias gráficas, de tamaño grande e interpretación directa y clara en el frente de los envases de BA. Los participantes destacaron el momento sociopolítico favorable local y regional que impulse la colaboración entre países para la implementación de esta medida, la existencia de experiencias regionales en curso, y destacaron la importancia de esta medida por ser considerada aquella que «disparará la implementación del resto de las intervenciones» al indicar qué productos debieran ser alcanzados por las otras intervenciones.

Las restricciones de las acciones de publicidad y *marketing* de las BA también fueron priorizadas en los tres grupos, a pesar de reconocer que la evidencia es escasa con relación al efecto de políticas de prohibición. Una preocupación relevante para los informantes de los tres grupos se relacionó con el alcance de las potenciales medidas de regulación y/o prohibición, sugiriendo que debería alcanzar no solo a medios de comunicación masiva, sino a Internet, aplicaciones móviles y redes sociales.

Finalmente, las medidas de modificación del entorno escolar solo fueron priorizadas por dos de los grupos; y las medidas educativas solamente por uno. Entre las razones para priorizar las medidas orientadas a modificar el entorno escolar, se mencionaron la «implementabilidad» a corto plazo, la importancia de la medida al responder a la Declaración de los Derechos de los niños y niñas, y el hecho de que ya se ha implementado en algunos países de la región, como Brasil, donde sólo se permite la comercialización de agua y jugos naturales.

### Barreras y facilitadores para la implementación de políticas públicas

En la Tabla 2 se presentan las barreras que fueron consideradas amenazantes por los tres grupos para la implementación de intervenciones para controlar el consumo de BA. Se identificaron cuatro tipos de barreras para las medidas generales: Incidencia política o «cabildeo» por parte de las industrias productoras de BA, el potencial rechazo social de las medidas, la dificultad en la articulación política y las dificultades en la implementación y el monitoreo. Además, se describen las barreras identificadas con relación a medidas específicas.

**Tabla 2:** Percepciones de los informantes clave acerca de las barreras que amenazan la implementación de intervenciones para controlar el consumo de bebidas azucaradas en Argentina, Brasil, El Salvador y Trinidad y Tobago.

Incidencia política o «cabildeo» por parte de las industrias productoras de bebidas azucaradas
Actividades de cabildeo con foco en congresistas que dificultan y/o retrasan la sanción de normas regulatorias.
Intereses privados de decisores políticos de no implementar medidas que afecten el financiamiento de sus campañas políticas.
Actividades de patrocinio a escuelas, o eventos deportivos donde participan niños, niñas y adolescentes.
Programas de responsabilidad social de las industrias productoras que influencian negativamente la opinión pública.
Promoción de programas de autorregulación de las industrias productoras a pesar de conocerse que tienen poco o nulo impacto.
Oposición de «partes interesadas» / rechazo social
Dueños de cafetines/quioscos; administración de escuelas y «cooperadoras», quienes se benefician de las ventas en escuelas.
Familias (sensación de «pérdida de control»). Falta la percepción de riesgo para la salud.
Percepción del consumidor de que ciertas medidas se implementan «para abaratar costos».
Resistencia popular al gravamen de ciertos productos de consumo masivo por desconfianza sobre el destino de la recaudación.
Percepción de que las economías regionales serán amenazadas / pérdida de empleos.
Presión potencial de las industrias que proveen insumos para la comercialización de las bebidas azucaradas.
Articulación política dificultosa
Relevante para los países federales, en los que es necesario que las jurisdicciones ratifiquen las normas legislativas nacionales.
Percepción de poca articulación interministerial, fundamentalmente entre Ministerios de Salud, de Hacienda/Economía, Desarrollo Social y Educación.
Poca transparencia en los procesos de toma de decisión en el ámbito de la política.
Desconocimiento desde los Ministerios de Salud sobre las influencias que actúan sobre los Ministerios de Hacienda/Economía.
Regulaciones sobre la comercialización regional de bebidas azucaradas, como el Mercado Común del Sur que son utilizadas por la industria como argumentos contra la modificación de las etiquetas para incluir advertencias gráficas.
Falta de apoyo por parte de sociedades científicas como actores dentro del sistema de salud.
Implementación, monitoreo y evaluación dificultosa
La comunicación pública es desafiante dada la percepción popular negativa de medidas de regulación por parte de los estados.
Falta de recursos económicos para garantizar el acceso público al agua potable en reemplazo de las bebidas azucaradas.
Falta de recursos económicos y/o humanos para monitorear y evaluar estas medidas.
Falta de evidencia científica que avale el beneficio clínico y económico de implementar estas medidas.
Disenso entre partes interesadas sobre los productos a los que aplicarían estas medidas.
Amenazas potenciales a medidas específicas
Estrategias de las industrias productoras para no trasladar al precio final los impuestos.
Potencial reducción del beneficio de los impuestos si no se considera la asequibilidad a largo plazo en contextos con inflación.
Demanda cruzada de productos si el alcance de las medidas es parcial (reemplazo de consumo por otro producto no saludable).
Multiplicidad y modernización ultrarrápida de las plataformas de comunicación y acceso a Internet dificultan la regulación y/o prohibición de la publicidad.

En relación con los facilitadores, para todas las intervenciones priorizadas se destacaron las experiencias exitosas de implementación en países de la región como Chile (etiquetado frontal) y México (impuestos); el hecho de que en algunos países ya se estén discutiendo proyectos legislativos para avanzar con algunas de estas intervenciones, la declaración política sobre la emergencia epidemiológica que representa la obesidad a nivel global y local, y la importancia que éste tópico ha tenido en la agenda política pública global; la experiencia positiva con algunas de estas medidas cuando se implementaron para reducir el consumo de tabaco, permitiendo la «reutilización» de los circuitos y procesos que permitieron el avance de estas medidas, facilitando la articulación multisectorial. En todos los casos se destacó el rol de las organizaciones civiles como motores sociales para promover estas intervenciones. Entre los actores relevantes que fueron mencionados se incluyen: sociedades científicas, organizaciones que promueven la incidencia política en temas relacionados con salud pública, organizaciones de derecho del consumidor, periodistas y comunicadores sociales.

### Necesidades de información de los decisores para promover políticas públicas

Al ser consultados sobre las necesidades de información hubo consenso entre los tres grupos de informantes clave en la necesidad de evidencia del impacto de estas intervenciones en términos de: a) prevalencia de obesidad y sobrepeso, diabetes, mortalidad y cantidad de años de vida perdidos prematuramente; b) impacto sanitario de los cánceres a los que se atribuye un mayor riesgo por el consumo de BA y de enfermedades cardiovasculares asociadas y c) costos para el sistema de salud. En relación con los subgrupos de población de interés, hubo consenso con la desagregación de estos resultados por sexo, grupos de edad y nivel socioeconómico. En el caso de los resultados económicos, mencionaron que desearían conocer los costos atribuibles por subsistema de salud, así como por evento de salud. Algunos grupos consignaron los siguientes resultados como relevantes: prevalencia de caries, *bullying*, aspectos relacionados con la calidad de vida, inequidades sanitarias y de género asociadas con la obesidad y sobrepeso, gastos en concepto de cuidado de salud que no son reembolsados, externalidades macroeconómicas, y uso de recursos sanitarios (hospitalizaciones, consultas ambulatorias, etc.).

### Disponibilidad y calidad de la información requerida para los modelos económicos y la definición de políticas

A través de un ejercicio deliberativo en grupos conformados por representantes de cada uno de los países del proyecto, se identificó la disponibilidad de información para el diseño del modelo de carga de enfermedad atribuible a consumo de BA y de evaluación de las intervenciones para reducir su consumo (Tabla 3). En general, hubo consenso en la disponibilidad de información para los parámetros demográficos básicos y los datos de consumo, aunque los participantes evaluaron una menor disponibilidad de información vinculada a la efectividad de las intervenciones para reducir el consumo de BA.

**Tabla 3:** Disponibilidad de fuentes de información por país.

Tipo de dato	Argentina	Brasil	El Salvador	Trinidad y Tobago
Factores de riesgo cardiovasculares	Sí	Sí	Sí	Sí
Consumo de bebidas azucaradas	Sí	Sí	A confirmar	Sí
Demografía	Sí	Sí	Probablemente	Sí
Epidemiología de enfermedades relacionadas	Parcial	Sí	Sí	No
Pérdida de productividad	Parcial	Sí	A confirmar	Desconocido
Caries	Sí	Desconocido	Desconocido	Desconocido
*Bullying*	Sí	Desconocido	Desconocido	Desconocido
Efectividad de potenciales intervenciones	No	Parcial	Parcial	No
Elasticidad precio de la demanda	Sí	Sí	No	No
Información impositiva	Parcial	Sí	A confirmar	No
Costos de eventos	Parcial	Sí	Sí	Sí
Costos de intervenciones	Parcial	Sí	No	No

### Grupos poblacionales vulnerables al consumo de bebidas azucaradas

Se destacó a lo largo de ambas jornadas la relevancia de considerar como grupo particularmente vulnerable a los niños, niñas y adolescentes. Uno de los principales motivos fue la relevancia de la instalación de hábitos alimentarios en este grupo etario y que es el motivo por el cual las empresas productoras de BA dirigen sus campañas de marketing hacia ellos, vulnerando sus derechos con repercusiones para el resto de su vida. Se destacó además la necesidad de obtener resultados desagregados por nivel socioeconómico para que se posibiliten estimaciones de equidad. 

Como observación general, cabe destacar que la perspectiva de género no fue introducida espontáneamente por los participantes como una dimensión a considerar en las dinámicas grupales ni en los plenarios. A fin de no inducir respuestas socialmente esperadas y/o políticamente correctas, se evitó preguntar utilizando explícitamente esta expresión («perspectiva de género» o «enfoque de género»), optando por indagar si las intervenciones a realizar o las informaciones a recoger deberían considerar grupos poblacionales diferenciales y, en tal caso, cuáles. Ante esta consulta, por lo general se priorizó la segmentación por edad (con particular interés por los niños) y por estrato socioeconómico, pero no por género. Uno de los grupos indicó que no hay demasiadas diferencias en el consumo de BA entre varones y mujeres, ni en obesidad, aunque quizás en esto último dan mayores indicadores las mujeres porque son quienes, en general, procuran en mayor medida atención médica. Ante otras preguntas más directas sobre especificidades de las mujeres (en vistas de la no respuesta espontánea), se registró que no eran percibidas como un grupo poblacional diferencialmente afectado.

## DISCUSIÓN

En este diálogo de políticas un grupo muy relevante de actores de la región, formado por decisores, académicos y miembros de organizaciones de la sociedad civil, lograron identificar y priorizar las políticas fiscales, el etiquetado gráfico frontal de alimentos y la regulación y/o prohibición de la publicidad, la promoción y el patrocinio de las BA como las principales intervenciones para disminuir su consumo. Las acciones educativas y la modificación del entorno escolar fueron planteadas como intervenciones relevantes, pero no prioritarias debido a la dificultad en su implementación. La principal barrera reportada para la implementación de las medidas destinadas a reducir el consumo de BA fue la interferencia por parte de las industrias productoras a través de diferentes estrategias como el cabildeo político retrasando o dificultando la sanción de normas regulatorias y la promoción de programas de responsabilidad social empresaria y de iniciativas de autorregulación. Esta percepción está alineada con múltiples ejemplos en la literatura de interferencia de la industria alimentaria para la implementación de políticas destinadas al control de enfermedades no transmisibles en Latinoamérica ^15^^-^^17^. Otras de las barreras destacadas fueron la dificultad en la implementación, monitoreo, evaluación y articulación de las políticas destinadas a reducir el consumo de BA y la necesidad de recursos para llevar estas actividades a cabo.

Hubo consenso entre los participantes de este diálogo de políticas de la relevancia de contar con información clara sobre la carga de enfermedad tanto en términos sanitarios como económicos y del impacto de las intervenciones destinadas a reducir el consumo de BA. En cuanto a la disponibilidad de información en Brasil, Argentina, El Salvador y Trinidad y Tobago para realizar modelos de estimación de carga de enfermedad asociada al consumo de BA y de las intervenciones para reducir su consumo, exceptuando por la categoría información demográfica, hubo una marcada heterogeneidad entre los países, lo que acrecienta aún más la relevancia de poder contar con estimaciones confiables.

Uno de los beneficios más importantes de los eventos de diálogos de política es el sentido de apropiación del tema por parte de los responsables de la toma de decisiones. Por otra parte, el diálogo, ya sea formal o informal, puede aumentar la confianza entre los responsables de la toma de decisiones, los investigadores y la sociedad civil, permitiendo el desarrollo de compromisos constructivos para la sociedad en su conjunto, anticipando las barreras y facilitadores para la implementación ^18^.

Este tipo de eventos toman relevancia para la formulación de políticas destinadas a combatir enfermedades no transmisibles ^19^^,^^20^. Podemos mencionar la experiencia en relación al consumo de tabaco en Latinoamérica, donde la existencia de múltiples diálogos de políticas a lo largo del tiempo permitió identificar y promover políticas exitosas en reducir la prevalencia de su consumo en los últimos años ^21^.

Al tratarse de un evento presencial, el número de participantes por país fue bajo, pudiendo no representar la totalidad de intereses con relación al consumo de BA dentro de cada país, si bien se realizó un esfuerzo especial por identificar a los líderes por país; particularmente en países grandes como Brasil o Argentina, diferentes actores subnacionales no estuvieron representados.

Este evento permitió un ambiente de discusión estructurado favoreciendo el intercambio entre los actores no sólo de un país en particular sino de los cuatro países que generarán información de carga de enfermedad e impacto económico del consumo de BA en las próximas etapas de este proyecto. El aporte regional brindado por representantes de organizaciones internacionales presentes en el debate aportó experiencias de otros países que promovieron este tipo de políticas, generando además una sensación de necesidad regional de avanzar en esta temática.

En conclusión, la realización de este diálogo de políticas permitió identificar las principales intervenciones de políticas públicas que resulta prioritario impulsar en Argentina, Brasil, El Salvador y Trinidad y Tobago para disminuir el consumo de bebidas azucaradas, identificando barreras y facilitadores, resultados esperados para promover su implementación, así como la información disponible y ausente para generar estos resultados.
